# Crystal and electronic structures of substituted halide perovskites based on density functional calculation and molecular dynamics

**DOI:** 10.1016/j.chemphys.2016.12.007

**Published:** 2017-03-01

**Authors:** Hiromitsu Takaba, Shou Kimura, Md. Khorshed Alam

**Affiliations:** Department of Environmental Chemistry and Chemical Engineering, School of Advanced Engineering, Kogakuin University, 2665-1 Nakano, Hachioji, Tokyo 192-0015, Japan

## Abstract

Durability of organo-lead halide perovskite are important issue for its practical application in a solar cells. In this study, using density functional theory (DFT) and molecular dynamics, we theoretically investigated a crystal structure, electronic structure, and ionic diffusivity of the partially substituted cubic MA_0.5_X_0.5_PbI_3_ (MA = CH_3_NH_3_^+^, X = NH_4_^+^ or (NH_2_)_2_CH^+^ or Cs^+^). Our calculation results indicate that a partial substitution of MA induces a lattice distortion, resulting in preventing MA or X from the diffusion between A sites in the perovskite. DFT calculations show that electronic structures of the investigated partially substituted perovskites were similar with that of MAPbI_3_, while their bandgaps slightly decrease compared to that of MAPbI_3._ Our results mean that partial substitution in halide perovskite is effective technique to suppress diffusion of intrinsic ions and tune the band gap.

## 1 Introduction

Recently, solar cells based on organic-inorganic lead halide perovskites become one of key photovoltaic materials [1], [2], [3], [4]. Kojima et al. reported that methyl ammonium lead iodide (MAPbI_3_, MA = CH_3_NH_3_^+^) based solar cells had high photovoltage of 0.96 V [5] and 3.8 % power conversion efficiency. Extensive efforts are continued to improve their efficiencies [6], [7], [8], [9] and within short time, solar cell based on MAPbI_3_ are increasing efficiencies more than 20% [10].

One of unique feature of hybrid organic-inorganic perovskite is a wide variation of composed materials, which is a combination of metal ions, halide ions, and organic molecules. Bernal et al. from first principles calculations show that introducing substitutional Br dopants for I anions in MASnI_3_ could facilitate charge transfer from the hybrid perovskite to the TiO_2_ electrode [11]. Replacement of Pb^2+^ with Sn^2+^ has already been successfully demonstrated, however the stability of these compounds is still an issue [12], [13]. Furthermore, partial substitution of Pb^2+^ by Sn^2+^ as well as Sr^2+^, Ca^2+^ and Cd^2+^, Bi-Ti based perovskites and chalcogenide perovskites have been explored both the theoretically and experimentally [14], [15], [16], [17]. Filip et al. reported computational screening of homovalent lead substitution, suggesting that Mg is a potential candidate for the partial replacement of Pb, which show that the band gap is tuneable over a range of 0.8 eV via the size of the cation at A position (A-site in the ABO_3_-type perovskite) [18]. Pellet et al. reported that a mixture of HNCHNH_3_^+^ and MA in the A position of the APbI_3_ leads to an enhanced short-circuit current [19]. These studies indicate the combinations of metal ions and organic ions give rise to enormous structural and electronically diversity, which provide the tuneable methodology of halide perovskites.

Stability of halide perovskite is a key factor for further development. Concerning a stability of perovskite solar cells, to gain an insight on the hysteresis in current–voltage curves, Haruyama et al. theoretically investigated diffusion barriers of anion and cations in tetragonal CH_3_NH_3_PbI_3_ and trigonal (NH_2_)_2_CHPbI_3_ [20]. Their calculated results indicated that the ion displacement can explain for the hysteresis in current–voltage curves and the suppression of ions diffusion would improve the stability of halide perovskite. Another studies implied that migration of ions is one of the major factor for aging perovskite solar cells [20], [21]. However, despite the extremely fast progress in perovskite-based solid state photovoltaics, the details of degradation mechanisms and stability of perovskite are not yet totally understood which is essential for their commercialization and wide-spread use.

**Fig. 1:**
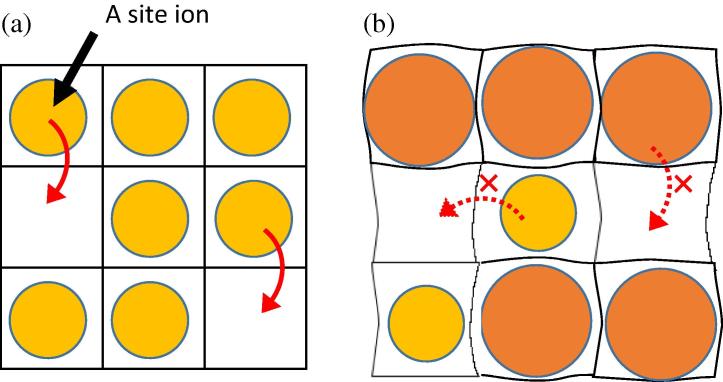
Schematics for the explanation of suppression effect of diffusion of ions by partially substituted perovskite. (a) The diffusion of A site ions between A positions in un-substituted perovskite. (b) The distorted structure introducing by partial substitution prevents the A site ions from the smooth migration between A positions.

In this work, to gain further insight into the durability of organig - halide perovskites, we theoretically investigate the statistic and dynamic properties of partially substituted halide perovskites in which MA are partially substituted by NH_4_^+^ or (NH_2_)_2_CH^+^ or Cs^+^ (= X). Here we mainly focus on suppression effect of diffusion of organic cations by partial substituting. Fig. 1 shows the schematic for the explanation of suppression effect of diffusion of A position ions (MA or X) by partially substituted perovskite. By mixing larger or smaller organic cations, the lattice would be distorted due to the mismatch of both ionic size and different interaction between A position ions and perovskite framework. Substituted larger cations can block a diffusion of smaller cations due to their slower diffusion rate, while the diffusion of larger cations is suppressed by distorted lattice structure. Here we report an effect of partially substituted CH_3_NH_3_^+^ perovskites on their bandgaps, structural parameters, and the diffusivity of cations, which would provide clue for developing highly durable perovskite solar cells.

## 2 Calculation method

### 2.1 Density functional theory (DFT)

Periodic DFT-GGA calculations using the PBE exchange correlation functional [22] were performed. DFT calculations were performed with CASTEP [23], [24], [25]. Electron-ion interactions were described by an ultrasoft pseudopotential. A 2 × 2 × 2 Monkhorst−Pack grid [26] in the Brillouin zone of the unit cell was employed for the geometry optimization, whereas a 5 × 5 × 5 Monkhorst−Pack grid was employed for the calculations of band structure. Kinetic cut off for plane-wave expansion of wave functions was 340 eV. A tolerance of self-consistent field was 2 × 10^−6^ eV/atom. To include the dispersion interactions in this class of systems, Tkatchenko-Scheffler (TS) scheme [27] was considered as a semi empirical dispersion-correction. A cubic MAPbI_3_ perovskite shows a crystal structure at room temperature. To investigate the stable structure and electronic properties of partially substituted perovskites by DFT, the unit cell composed of 2 × 2 × 2 time of a unit cell of the cubic CH_3_NH_3_PbI_3_ was considered. An initial lattice parameter of cubic CH_3_NH_3_PbI_3_ before geometry optimization was *a* = 12.66 Å which was experimentally reported [28].

### 2.2 Molecular dynamics (MD)

MD simulations were performed with program package Forcite [29]. UNIVERSAL force field [30] was used as a force field representing atomic interaction in all MD calculations. Several types of force field were tested whether they reproduce experimentally reported crystal structure of CH_3_NH_3_PbI_3_. UNIVERSAL force filed was confirmed to reproduce a cubic structure of CH_3_NH_3_PbI_3_. The initial atomic position and cell sizes of investigated unit cell were determined from the results of the geometry optimization calculations by DFT. All MD simulations performed with canonical ensemble (NVT). Temperature was controlled at 300 K by Berendsen’s method [31]. Periodic boundary conditions were employed and electrostatic interactions were calculated using the Ewald summation method [32]. The MD unit cell composed of 4 × 4 × 4 time of the unit cell. The lengths and angles of the unit cell are determined from the DFT calculation results, which are shown in Table 1. The diffusion coefficient of each ion is determined based on the time evolution of the mean square displacements (MSD) using the Einstein’s equation. This quantity can be determined by recording the atomic positions regularly during the simulation. In order to obtain the reliable diffusion coefficient, the larger time steps are required for the MD calculation. Thus, total time steps for one run are 2 × 10^6^ steps (1 step = 1.0 fs). Diffusion coefficients were estimated using the linear part of slopes of MSD where the system reaches at equilibrium. For most of MA, the MSD data after 500 ps was used to determine diffusion coefficients.

## 3 Results and discussion

To study structural and electronic properties for partial substitution of organic cation MA, half number of MA in the unit cell was partially replaced by the NH_4_^+^ or (NH_2_)_2_CH^+^ or Cs^+^. The calculated Goldshmidt tolerance [33], *t*, and the ionic size of considered cation species are shown in Table 2. These *t* values indicate that considered cations can compose to a perovskite structure. Larger value of *t* indicates more distorted perovskites. Therefore, a combination of two kinds of cation having different *t* values for substitution at A position is expected to introduce a distortion to the lattice keeping a perovskite structure.

To check the effect of dispersion interactions, we performed selected calculations for this super cell using TS scheme. As shown in Table 3, lattice parameters of the optimized super cell from the calculation without dispersion correction were *a* = 12.87 Å, *b* = 12.88 Å, and *c* = 13.03 Å with the volume of 2149 Å^3^. Lattice parameters of the optimized structure from the calculation with dispersion correction were *a* = 12.66 Å, *b* = 12.68 Å and *c* = 12.82 Å with the volume of 2057 Å^3^. The calculation with dispersion correction gives acceptable lattice parameters those are closer to the initial cubic lattice of *a* = 12.66 Å. The calculated bandgap with considering dispersion force is close to the experimental value of 1.55 eV [5]. Improvement of band gap estimation comparing with the experimental values by considering dispersion force was discussed in the previous work [36] in which the similar trend to our results was reported. Because of the accuracy in the calculated bandgap and structure, TS dispersion correction were considered in the following calculations.

**Fig. 2:**
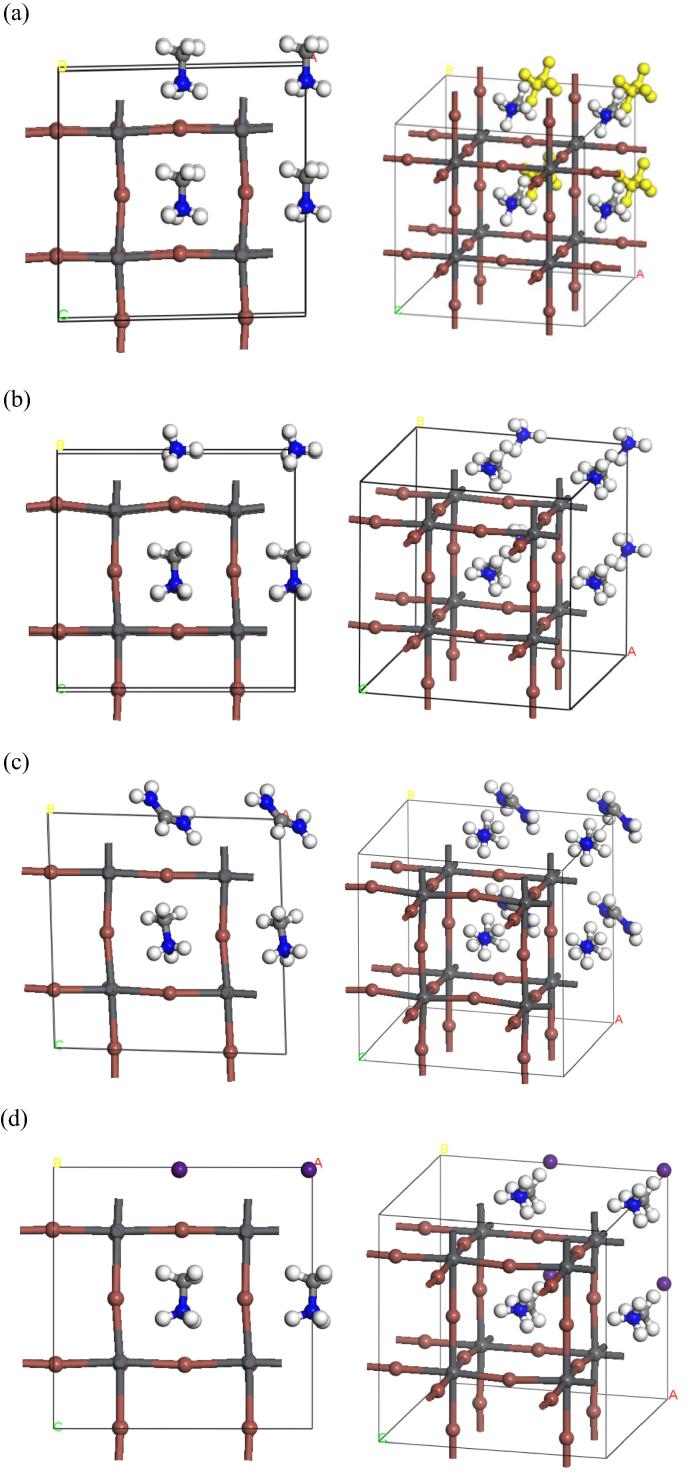
(a) Optimized structures of cubic MAPbI_3_. 4 yellow colored MA molecules indicate the substituted positions in (b)–(d) models. (b) the optimized structure for partial substitution of MAPbI_3_ by NH_4_^+^, (c) the optimized structure for partial substitution of MAPbI_3_ by (NH_2_)_2_CH^+^, and (d) the optimized structure for partial substitution of MAPbI_3_ by Cs^+^. In (a)–(d), right and left figures show different angles. (For interpretation of the references to color in this figure legend, the reader is referred to the web version of this article.)

We partially replaced the organic cation with the different substituents (NH_4_^+^, (NH_2_)_2_CH^+^ and Cs^+^) as shown in Fig. 2. In all the cases, we have inserted 4 ions as substituents into the unit cell with maintaining 4 molecules of MA at their initial position. Several possible distributions of partially substituted cations can be considered. In this study, four molecules were substituted those located at the *ab*-axis plane as shown in Fig. 2, because this lamellar like distribution would introduce large structural distortion compared to that of randomly substituted structures. The calculated lattice parameters, angles, and the cell volume of the partially substituted structures by DFT are summarized in Table 1. The cell volumes of partially substituted perovskite changed compare to that of MAPbI_3_. The cell volume increases with the increase in the ionic radius of substituted organic molecules. It is noted that the tendency of the cell volume is different for inorganic substituent, Cs^+^. This may be because Cs^+^ has a unique interaction to perovskite framework compared to the interaction of organic cations. Table 1 shows that the lattice angles of partial substitution perovskites slightly differ from those of un-substituted perovskite. It is noted that most distorted structure is ((NH_2_)_2_CH^+^)_0.5_MA_0.5_PbI_3_ of which the ionic radius of cations is largest among other systems. This means that the partial substitution of organo-lead halide perovskite can vary a space within perovskite framework, which would change mobility of ions at A position.

**Fig. 3:**
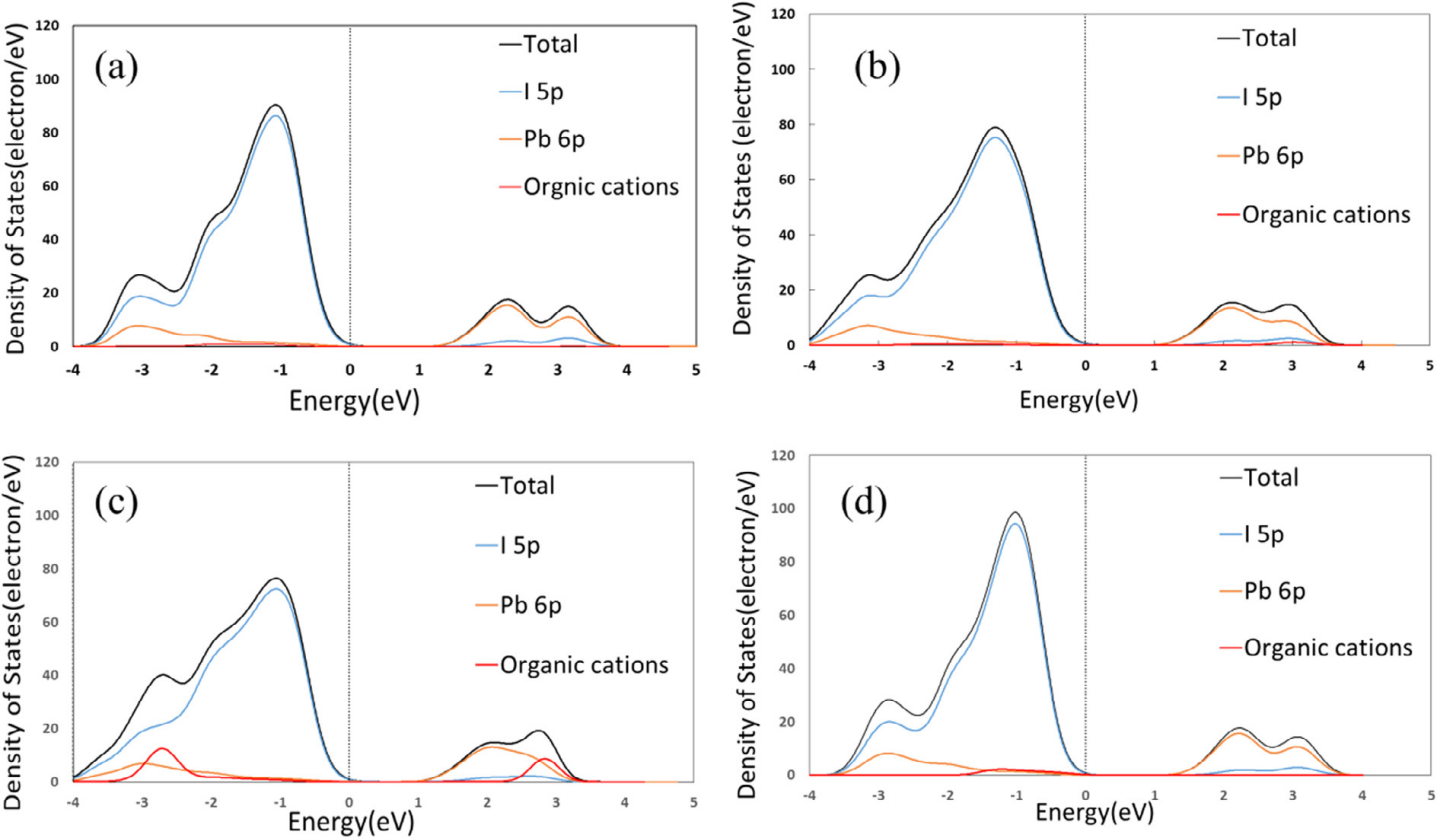
Calculated partial density of states (PDOS) of (a) CH_3_NH_3_PbI_3_, (b) partially substituted MAPbI_3_ by NH_4_^+^, (c) partially substituted MAPbI_3_ by (NH_2_)_2_CH^+^, and (d) partially substituted MAPbI_3_ by Cs^+^.

In Fig. 3(a) depicts partial density of states (PDOS) of the MAPbI_3._ From the PDOS of the MAPbI_3_, the valence and conduction bands consist mostly of the I 5p and Pb 6p orbitals, respectively, which is consistent with previous report [37]. Fig. 3(b–d) show the PDOS of partially substituted perovskites. These figures illustrates that there are no significant differences in the electronic structures between partially substituted MAPbI_3_ and MAPbI_3_. Usually, the organic cation acts as a charge compensating center but does not participate in the frontier electronic and band structure [11], [38], [39], [40]. That is true for our investigated partially substituted MAPbI_3_ perovskites. However, the calculated band gap of partially substituted perovskites, which are summarized in Table 1, are slightly smaller than that of MAPbI_3_.

**Fig. 4:**
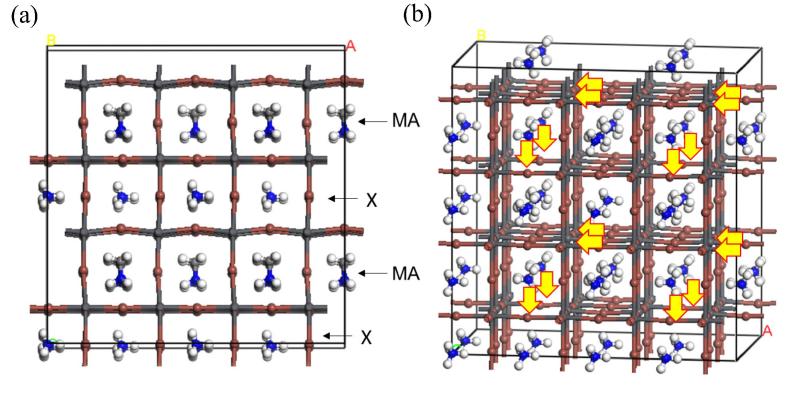
(a) initial structure of a unit cell of partially substituted MAPbI_3_ by NH_4_^+^ used in MD simulations. (b) same initial structure with arrows that indicate the positions of defects of I^-^.

To investigate the effect of a lattice distortion on ionic mobility, MD simulation using the partially substituted perovskite structure was conducted. The initial structure of the unit cell of partially substituted MAPbI_3_ by NH_4_^+^ was shown in Fig. 4(a) where MA and substitute X are layered. For halide perovskites, it is considered that vacancy migrations seem most dominant for ion diffusion processes [20]. Actually, no migration of cation atoms was observed in the 2 ns of MD calculation for the model of MAPbI_3_ with no defect. Therefore, we introduced 25% of vacancy at A position to the unit cell. In the unit cell, 16 atoms of I^-^ were also removed for charge compensation, which resulting in the chemical formula of MA_0.375_X_0.375_PbI_2.75_ (X = NH_4_^+^, (NH_2_)_2_CH^+^, and Cs^+^). The vacancy concentration of our models is 2 × 10^21^ cm^−3^, which is reasonably comparable to the predicted vacancy concentration of 10^17^–10^20^ cm^−3^ for charged Pb^2+^, I^−^, and MA^+^ within the assumption of thermal equilibrium and noninteracting defects [41]. The positions of anion vacancies were indicated in Fig. 4(b), which are distributed over the unit cell. The vacancies of counter cation were located near the vacancies of I^−^.

**Fig. 5:**
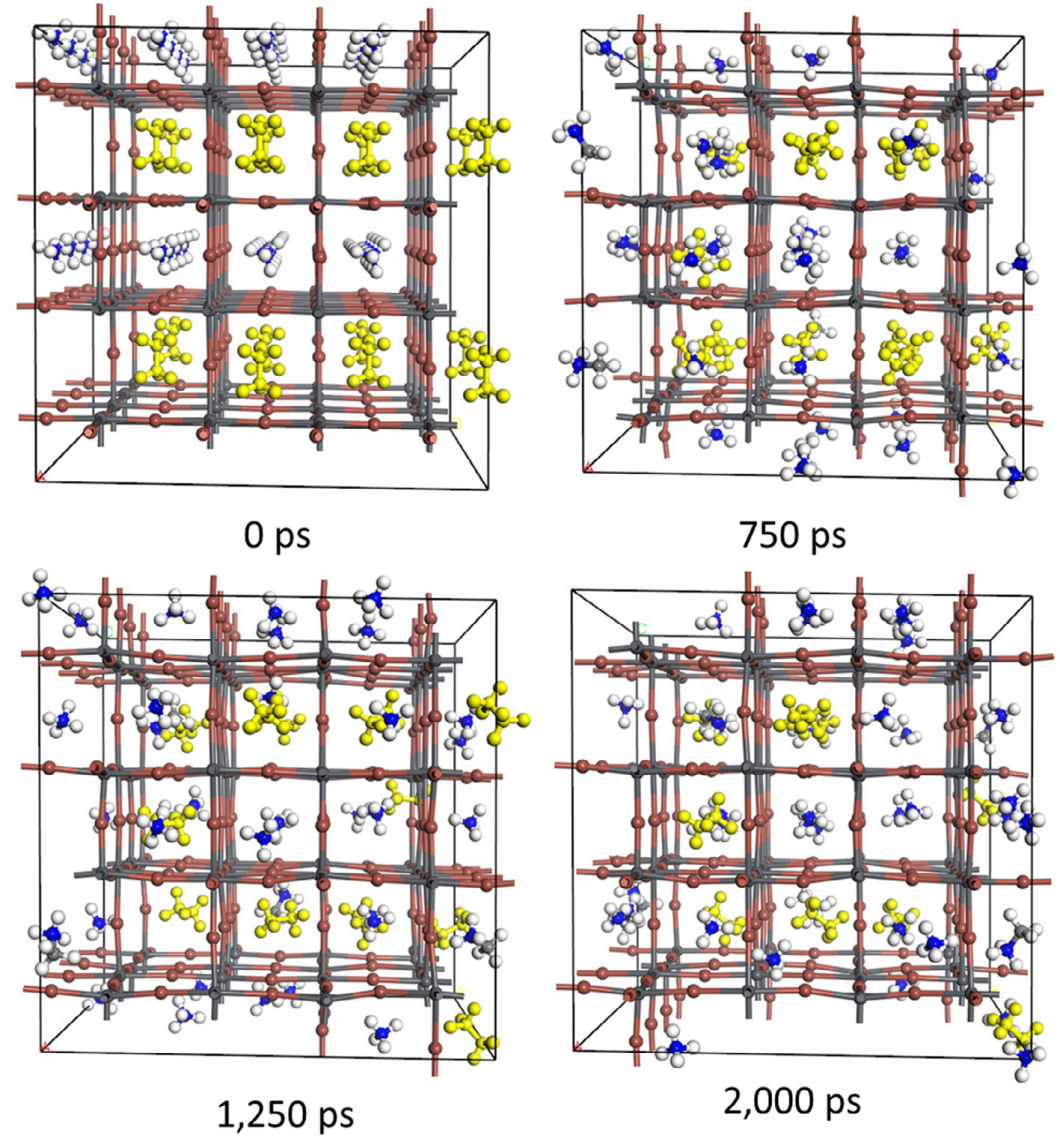
The snapshots of the unit cell obtained from MD for MA_0.375_(NH_4_)_0.375_PbI_2.75_ at different time. Yellow colored molecules represents the MA molecules. (For interpretation of the references to color in this figure legend, the reader is referred to the web version of this article.)

**Fig. 6:**
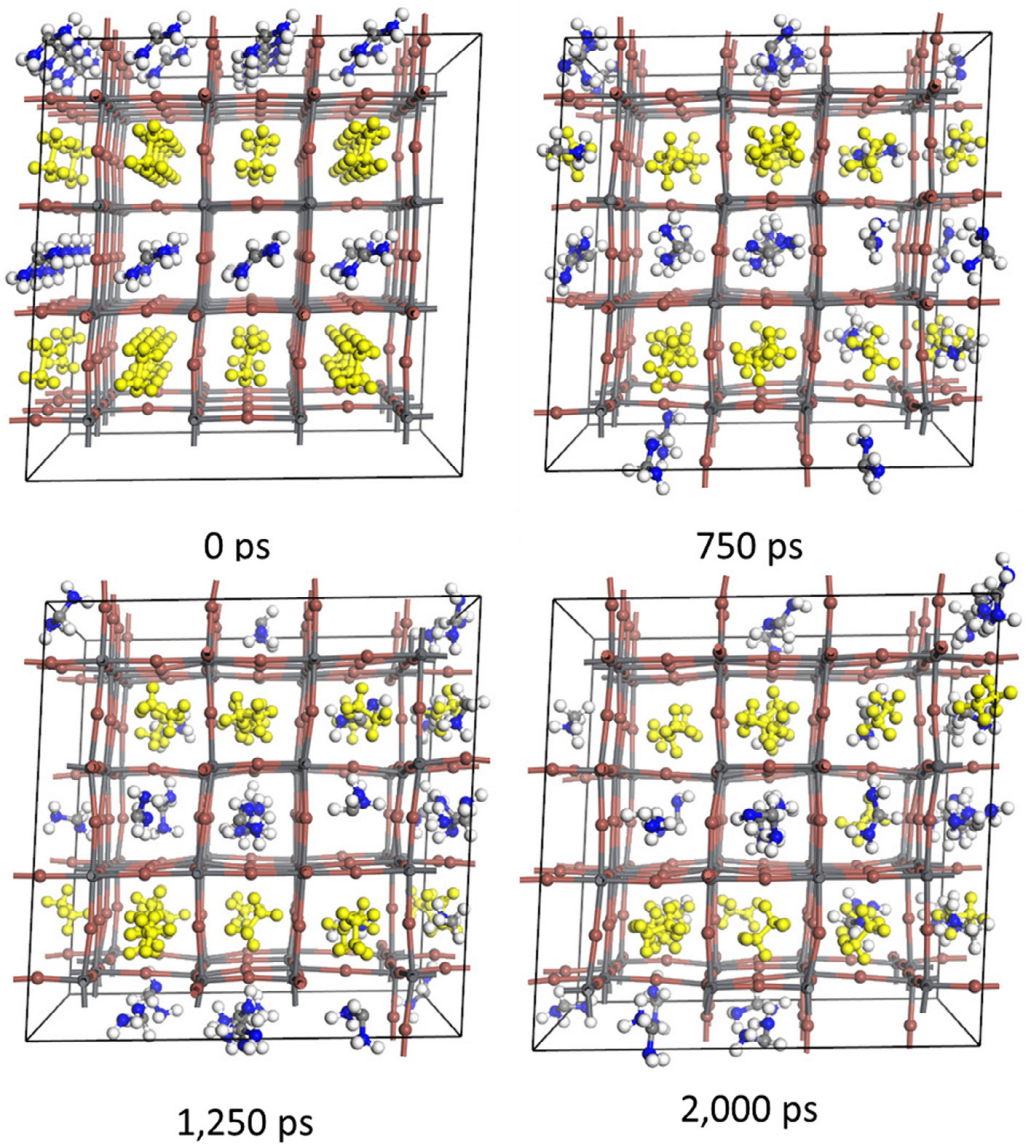
The snapshots of the unit cell obtained from MD for MA_0.375_((NH_2_)_2_CH)_0.375_PbI_2.75_ at different time. Yellow colored molecules represents the MA molecules. (For interpretation of the references to color in this figure legend, the reader is referred to the web version of this article.)

Fig. 5 shows snapshots of the structure of the unit cell obtained from MD for MA_0.375_(NH_4_)_0.375_PbI_2.75_. As shown in this figure, the migration of MA and NH_4_^+^ between A positions is clearly indicated with maintaining perovskite frame structure. The calculated diffusion coefficients of cations at A positions are summarized in Table 4 (The time evolution of MSD are show in the Supporting information). It is noted that the absolute value of diffusion coefficient depends on adapted structural model and force filed type. Since the reproducibility of diffusion coefficients by our used force filed is not guaranteed, we discuss here the relative comparison of the diffusion coefficients for different models. Although the size of the unit cell of partially substituted perovskite by NH_4_^+^ shrunk compared to un-substituted one, the averaged diffusion coefficient in MA_0.375_(NH_4_)_0.375_PbI_2.75_ increases compared to that for un-substituted one. This is because the smaller cation of NH_4_^+^ promotes a migration of MA by collisions or prompt supplying vacancy for MA migration. Table 4 also shows that the diffusion coefficient of MA in MA_0.375_Cs_0.375_PbI_2.75_ significantly increases compared to that of MA_0.75_PbI_2.75_. This is because of the swelling of the unit cell resulting in extending the diffusion space promotes the migration of MA molecules. Fig. 6 shows the snapshots of the unit cell obtained from MD at different times for MA_0.375_(NH_4_)_0.375_PbI_2.75_. With a comparison to Fig. 5, the migration of MA between A positions is obviously suppressed. From Table 4, the diffusivities of averaged and both cations in MA_0.375_ (NH_2_)_2_CH _0.375_PbI_2.75_ are smaller than those in MAPbI_3_ and also in other substituted systems. This would be because of the effect of distortion of the lattice, where larger cations inhibit diffusion of smaller cations while a distortion caused by the mismatch of lattice suppress diffusion of larger cations. This result implies that fabrication of partially substituted halide perovskites could suppress migration of cations at A position.

## 4 Conclusion

We investigated the structural, electronic and ionic diffusivities of the partially substituted cubic MA_0.5_X_0.5_PbI_3_ (X = NH_4_^+^, (NH_2_)_2_CH^+^, and Cs^+^) by density functional theory and molecular dynamics. The electronic structure analysis indicated that bandgaps of the investigated partially substituted perovskite are slightly lower for all the cases compared to the MAPbI_3_ perovskite. Calculated bandgap decreased with increasing the substituted cation size. MD results show that the mobility of the averaged cations in partially substituted systems depends on the size of cations. The diffusivities of cations in partially (NH_2_)_2_CH^+^ substituted MAPbI_3_ is significantly suppressed comparing to that of un-substituted and also the other substituted systems. In our simulations, the migration of cations at A position dominantly occur, although all atoms are allowed thermally to move, because we adapted the bonding potential for the bonds between I^−^ and Pb^2+^, which mostly prohibits a bond dissociation and reformation of Pb-I. Haruyama et al. theoretically reported that both cation and anion can diffuse in the lattice and the activation barrier of I^−^ is ca. 0.45 eV that is less than that of MA^+^ [20]. Therefore, the migration of I^−^ may influence the cation migration, which needs further investigation. In summary, this computational study shows that the partially substitution of the organic cation with the larger cation can suppress the mobility of the ions at A position and may contribute to increase a durability of halide perovskite solar cells.

## Figures and Tables

**Table 1:** The optimized structural parameters of partially substituted MAPbI_3_ by DFT with dispersion correction.

MA_0.5_X_0.5_PbI_3_	*α, β, γ* (°)	cell volume (Å^3^)	band gap (eV)	Lattice constants a, b, c (Å)
X = NH_4_^+^	90.97	2025	1.35	12.67
	90.02			12.61
	90.85			12.68
X = (NH_2_)_2_CH^+^	90.84	2099	1.32	12.92
	87.82			12.63
	89.28			12.87
X = Cs^+^	90.07	2137	1.53	12.85
	89.96			12.82
	90.22			12.97

**Table 2:** Ionic radii and calculated Goldschmidt tolerance factors, *t*, of considered ions at A position.

Ion	Ionic radius (Å)	*t*
CH_3_NH_4_^+^	2.17 [34]	0.875
NH_4_^+^	1.46 [34]	0.733
(NH_2_)_2_CH^+^	2.53 [34]	0.947
Cs^+^	1.67 [35]	0.775

**Table 3:** Lattice parameters of the optimized structure of MAPbI_3_ by DFT with and without dispersion correction.

	Lattice constant a, b, c (Å)	*α,β,γ* (°)	Cell volume (Å^3^)	Band gap (eV)
Without dispersion correction	12.87	86.74	2149	1.63
	12.88	93.31		
	13.03	86.91		
With dispersion correction	12.66	89.14	2057	1.56
	12.68	91.23		
	12.82	89.76		

**Table 4:** Calculated diffusion coefficients (*D*) for cations in MAPbI_3_ and partially substituted perovskites with vacancies (MA_0.375_X_0.375_PbI_2.75_).

*D* (×10^−11^ m^2^ s^−1^)	Un-substituted perovskite	Partially substituted perovskites		
		X = NH_4_^+^	X = (NH_2_)_2_CH^+^	X = Cs^+^
Averaged *D*	1.17	2.18	0.683	4.35
*D* of MA	1.17	2.35	1.02	6.78
*D* of X	–	1.90	0.35	1.92
