# Supplementary material for: Crystal and electronic structures of substituted halide perovskites based on density functional calculation and molecular dynamics
Source: Chem Phys. 2017 Mar 1;485-486:22–8. doi: 10.1016/j.chemphys.2016.12.007 (PMC5364368; doi:10.1016/j.chemphys.2016.12.007)
Supplement: Supplementary data [file mmc1.docx]

Supporting information

Crystal and electronic structures of substituted halide perovskites based on density functional calculation and molecular dynamics

Hiromitsu Takaba^*^, Shou Kimura and Md. Khorshed Alam

Department of Environmental Chemistry and Chemical Engineering, School of Advanced Engineering, Kogakuin University, 2665-1 Nakano, Hachioji, Tokyo 192-0015, Japan

Fig. 1s Time evolution of MSD for MAPbI_3_ and partially substituted MA_0.5_X_0.5_PbI_3_ perovskites models (X = NH_4_^+^_,_ (NH_2_)_2_CH^+^ and Cs^+^).
